# Effects of Greenselect Phytosome® on weight maintenance after weight loss in obese women: a randomized placebo-controlled study

**DOI:** 10.1186/s12906-016-1214-x

**Published:** 2016-07-22

**Authors:** Luisa Gilardini, Lucia Pasqualinotto, Francesco Di Pierro, Paolo Risso, Cecilia Invitti

**Affiliations:** Department of Medical Sciences and Rehabilitation, IRCCS Istituto Auxologico Italiano, Via Ariosto 13, 20145 Milan, Italy; Scientific Department, Velleja Research, Milan, Italy; Department of Health Sciences (DISSAL), University of Genoa, Genoa, Italy

**Keywords:** Green tea, Weight maintenance, Obesity, Fat mass

## Abstract

**Background:**

Most subjects regain weight after weight loss due to compensatory adaptations finalized to maintain stable body energy stores. Green tea (GT) preparations, which help maintain energy expenditure while dieting could be a useful strategy to facilitate weight maintenance. The usefulness of GT preparations in weight maintenance has been poorly studied so far with conflicting results. This study evaluated if a supplement of GSP and piperine helps obese women to maintain the weight loss obtained with a 3-month lifestyle intervention.

**Methods:**

In a randomized placebo-controlled study, we examined whether a highly bioavailable GT extract may counteract weight regain after weight loss. Forty obese women (age 50.1 ± 10.1 years, Body Mass Index (BMI) 36.3 ± 2.7 kg/m^2^) underwent a 3-month lifestyle intervention. At the end of the intervention, the women were randomized in two groups for the weight-maintenance phase: 20 of them were prescribed twice a day, for 3 months, with a formula containing 150 mg/dose of Greenselect Phytosome® and 15 mg/dose of pure piperine (GSP group), and 20 were given placebo (P group). Anthropometric measures and body composition were measured before (V-3) and after lifestyle intervention (V0), 1 (V1), 2 (V2), and 3 (V3) months after prescribing supplements and 3 months following the discontinuation of supplements (V6).

**Results:**

Lifestyle intervention induced a significant weight reduction in both groups with similar weight change (−6.2 ± 2.6 in GSP group vs. −4.8 ± 3.1 % in P group). In the GSP group, V1 in comparison to V0, had further reduction in weight and fat mass, which remained stable at V2 and V3 and increased at V6. In the P group, weight and fat mass increased from V2 onwards. Weight changes in GSP group and P group from V0 to V3 were −1.0 kg (95 % CI −2.5 to +0.5) and + 0.3 kg (95 % CI −0.9 to +1.6), respectively.

The proportion of women with weight loss ≥ 5 % was greater in the GSP group than in the P group (75 % vs. 45 % at V1, and 60 % vs. 30 % at V6, *p* < 0.05 for both groups).

**Conclusions:**

Greenselect Phytosome® devoid of caffeine may have a clinical potential for the maintenance of weight after intentional weight loss.

**Trial registration:**

Clinicaltrials.gov NCT02542449 (September 2015)

**Electronic supplementary material:**

The online version of this article (doi:10.1186/s12906-016-1214-x) contains supplementary material, which is available to authorized users.

## Background

Genetic, epidemiological, and physiological studies indicate that body weight is highly regulated, and the increasing prevalence of obesity reflects the interactions of genes favoring energy conservation and storage in an environment that enables access to food calories and a more sedentary lifestyle. Weight loss can be achieved by reducing energy intake and at the same time sustaining energy expenditure. Although weight loss interventions usually lead to weight loss shortly after intervention, majority of subjects once again regain weight after the intervention due to compensatory adaptations finalized to maintain stable body energy stores. The physiological adaptation to weight loss involves several biological pathways mediating the utilization and storage of energy and the regulation of appetite [1]. In both, lean and obese individuals, maintenance of a 10 % or greater reduction in body weight is associated with a decline in 24-hour energy expenditure of approximately 20–25 % [2]. The restraint of the decline in energy expenditure while dieting along with GT preparations could be a useful strategy to facilitate weight maintenance. GT contains a complex mixture of polyphenolic compounds belonging to the family of catechins, mainly epigallocatechin gallate, which are responsible for most of the pharmacological activity of GT. The principal mechanisms of GT are stimulation of fat oxidation through up-regulation of lipid-metabolizing enzymes and an increase in norepinephrine levels and energy expenditure through inhibition of catechol-O-methyltransferase [3].

The ability of GT preparations assisting weight loss was evaluated in a Cochrane Systematic Review that included 14 randomized controlled trials [4]. This review concluded that the weight loss produced by GT preparations is unlikely to be clinically relevant since it was not statistically significant in majority of the studies. However, the usefulness of GT preparations in weight maintenance has been poorly studied so far with conflicting results [4].

Despite the wide “nutraceutical” use of GT, pharmacological and clinical data show that these molecules are poorly absorbed orally. Formulation with phospholipids (Phytosome strategy) however, has shown increased absorption with some classes of natural products, including polyphenolics. Thus, the absorption of Greenselect Phytosome® (GSP) is about 3-fold higher compared to the unformulated extract [5], and GSP was also shown to reduce body weight in subjects with obesity and metabolic syndrome [6, 7]. Currently, a new formulation of GSP combined with piperine, a thermogenic agent, has been developed. This new formulation increases the absorption of various phenolics [8] and enhances the pharmacodynamics of GSP. This study evaluated whether dietary supplements of GSP and piperine help obese women to maintain the weight loss obtained with a 3-month lifestyle intervention.

## Methods

### Study participants

The study sample consisted of 40 obese Caucasian women who were recruited from those patients referred to the IRCCS Istituto Auxologico Italiano for a weight-loss lifestyle intervention. Women with uncontrolled hypertension and history of cardiovascular or cerebrovascular events were excluded from the study. Sample size was calculated assuming a ≥5 % weight loss would be the outcome obtained with 3 months of lifestyle intervention. The effective size of the study group was calculated for a binomial test irrespective of weight loss ≥5 %, looking for at least a difference of 25 % between the proportion of women reaching this goal in the placebo and treatment group. We designed our trial with an alpha error of 5 % and a power of 95 %. Calculation was performed using G*Power 3.1, obtaining a sample size of 42 subjects, that was rounded to 20 women for each group.

The Ethics Committee of the Istituto Auxologico Italiano approved the study, and all subjects gave their informed consent after we provided a full explanation of the study.

### Intervention

**Fig. 1:**
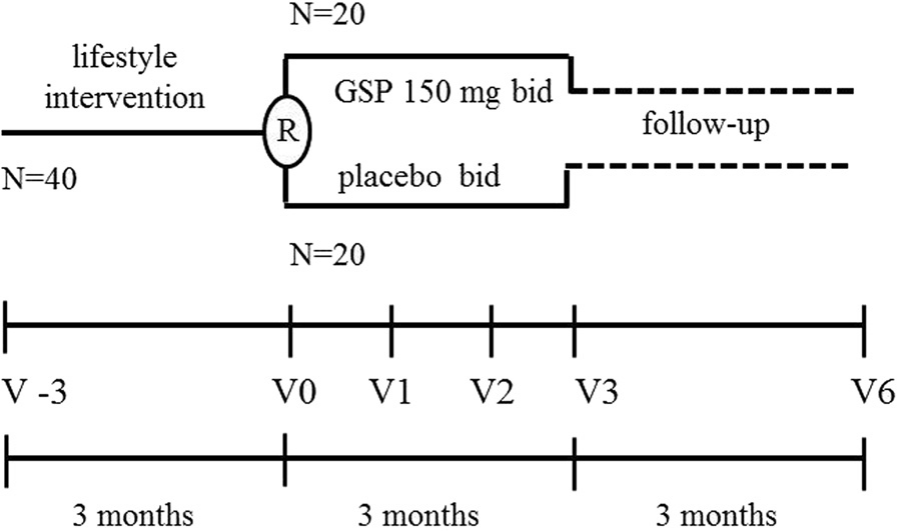
Study design

All obese women completed a 3-month lifestyle intervention. At the end of the intervention, women were randomly assigned to two groups for the weight-maintenance phase: 20 of them were prescribed supplements twice a day for 3 months and were named GSP group. This included Globes® (Pharmextracta, Pontenure, Piacenza, Italy), an enteric coated formulation containing 150 mg/dose of Greenselect Phytosome®. Greenselect Phytosome® is a highly standardized extract of *Camellia sinensis*, titrated as > 60 % polyphenols and > 40 % in epigallocatechin-O-gallate, complexed with soy distearoylphosphatidylcholine, and pure piperine (15 mg/dose) from *Piper nigrum L.* The remaining 20 were designated as the P group and received placebo twice a day for 3 months, which was undistinguishable from the active in terms of size, shape, taste, odor, primary and secondary packaging. Tested dietary supplements and placebo were both manufactured in S.I.I.T. srl (Trezzano S/N, Milan, Italy). Randomization was done using the sealed envelope system and compliance was checked by counting the left-over and returned capsules. The whole trial was conducted in blind, including the subjects and the dietician who collected anthropometric measures and assessed the adherence to diet during dietary supplements and follow-up visits. All women attended a monthly clinical visit while they were taking dietary supplements and 3 months after the end of the supplement intake (Fig. 1).

Lifestyle intervention consisted of weekly individual sessions for nutritional education, advice reinforcement on exercise activity and peer group psychological support. A self-monitor diary, which included details of food consumption, daily physical activity and emotional reactions, was used as a tool for education and reinforcement. Daily caloric requirement was calculated using the Harris-Benedict equation and an individual activity factor. A diet based on a 500-kcal/d deficit from the individual estimated caloric requirement was prescribed. The diet, which was high in vegetables, low in salt and simple sugars consisted of 25 % of total energy intake as protein, 20 % as fat and 55 % as carbohydrate. Fresh foods, at least three fish meals per week and avoiding alcohol, were recommended. The prescribed physical activity program was 210 min per week consisting of 70 % moderate-intensity aerobic physical activity and 30 % muscle-strengthening activities. The patient’s dietary compliance and the average weekly level of physical activity were recorded at each session. Diet history and levels of physical activity were collected before and after the 3-month lifestyle intervention. Food energy and nutrient intake was calculated using a computerized database and analysis program (Dietosystem version 3.0; DS Medica S.r.l., Milan, Italy). Physical activity was assessed using the short version of the International physical activity questionnaire (IPAQ) that computes the Metabolic Equivalent (MET: time spent in physical activity, expressed in minutes per week) [9].

At the end of the 3-month lifestyle intervention, women were encouraged to continue with the same diet and physical activity program recommended during lifestyle intervention.

### Outcome measures

The primary outcome was the proportion of women with ≥ 5 % weight loss during dietary supplements and three months after their discontinuation in GSP and placebo group.

The secondary outcomes were the changes in weight and fat mass during the intake of dietary supplements and three months after their discontinuation in GSP and placebo group.

Further outcomes were changes in blood pressure and heart rate during the intake of dietary supplements and three months after their discontinuation in GSP and placebo group.

### Measurements

Anthropometric measures, body composition, blood pressure (BP) and heart rate were measured at V-3, V0, V1, V2, V3, and V6. Anthropometry was measured using a body composition analyzer, which measures weight, height and Body Mass Index (BMI) with joined function of body fat analysis (Biki 300, Jawon Medical, Korea). Waist circumference was measured at the level of the umbilicus. At V0, V3 and V6, the women’s adherence to the diet was recorded by the dietician using a short questionnaire (9 items) that provided a score ranging from 0 to 18, where lower values indicate better adherence (Additional file 1). Three BP measurements separated by 5 min were obtained in sitting position at each visit and mean values were used for the analysis.

### Statistical analysis

Analysis of variance was used to compare differences among groups. Frequencies were compared using a *χ*^2^ test. Paired *t*-test was used to compare a) differences between variables at V0 vs. V-3 in each group (Table 1), b) differences between weight and fat mass at V0 vs. V-3, at V1 vs. V0, at V2 vs. V1, at V3 vs. V2 and at V6 vs. V3 (Fig. 3). Logistic regression analysis was used to evaluate the probability to have a weight reduction ≥5 % with Globes® with respect to placebo. A probability value < 0.05 was considered significant. Data are given as the means ± SD. All analyses were performed using SPSS version 22.0 (SPSS, Chicago, IL, US).

**Table 1:** Characteristics of obese women of GSP and P group before and after the 3-month lifestyle intervention

	GSP group (*n* = 20)		P group (*n* = 20)	
	V-3	V0	V-3	V0
Age, years	47.6 ± 10.3	-	52.6 ± 9.6	-
Hypertension, %	10	-	35	-
Diabetes, %	5	-	15	-
Weight, kg	94.3 ± 9.8	88.4 ± 9.1**	90.6 ± 6.6	86.3 ± 5.6**
Waist circumference, cm	112.5 ± 10.0	107.1 ± 10.1**	113.9 ± 5.4	110.3 ± 5.7**
Fat mass, kg	43.0 ± 5.8	40.9 ± 5.7**	41.9 ± 3.5	40.7 ± 3.5*
Fat mass/soft lean mass, ratio	0.98 ± 0.10	0.96 ± 0.11	1.0 ± 0.09	0.99 ± 0.08
Systolic BP, mmHg	126.0 ± 13.9	120.5 ± 10.7*	123.7 ± 10.9	119.2 ± 8.6
Diastolic BP, mmHg	79.0 ± 6.4	77.2 ± 4.7	79.5 ± 6.8	77.5 ± 5.3
Heart rate, beats/min	73.8 ± 8.6	73.1 ± 6.9	73.4 ± 9.1	70.7 ± 6.7*

V-3: before lifestyle intervention. V0: after the 3-month lifestyle intervention-start of dietary supplements. *p < 0.05, **p < 0.0001 compared to V-3. Comparisons between GSP and P group were NS at –V3 and at V0

## Results

**Fig. 2:**
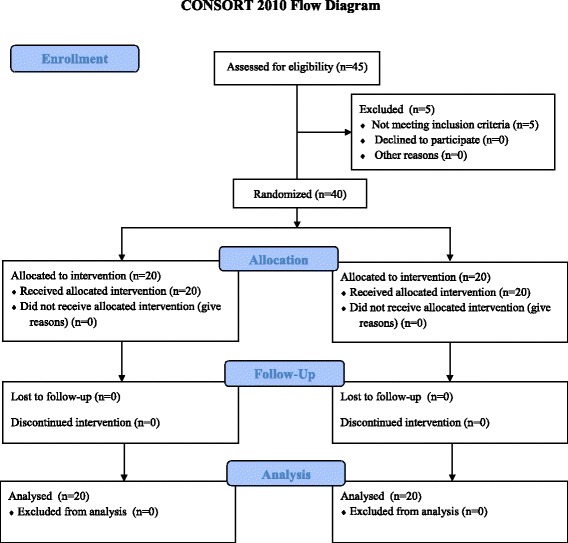
Flow of patients in the study

The adherence to dietary supplements was complete in both groups and all subjects completed the study. The flow of patients in the study is shown in Fig. 2. Table 1 shows the characteristics of obese women belonging to GSP and P groups before (V-3) and after the 3-month lifestyle intervention (V0). No differences were observed between the two groups at V-3 except for the energy intake that was greater in the GSP group than in the P group. At V0, the energy intake decreased and physical activity increased in both groups (Additional file 2). The intervention induced a significant weight reduction in both groups with similar weight changes (−6.2 ± 2.6 in GSP group vs. −4.8 ± 3.1 % in P group, NS). At V0 anthropometric measures, BP and heart rate were similar in both groups.

**Fig. 3:**
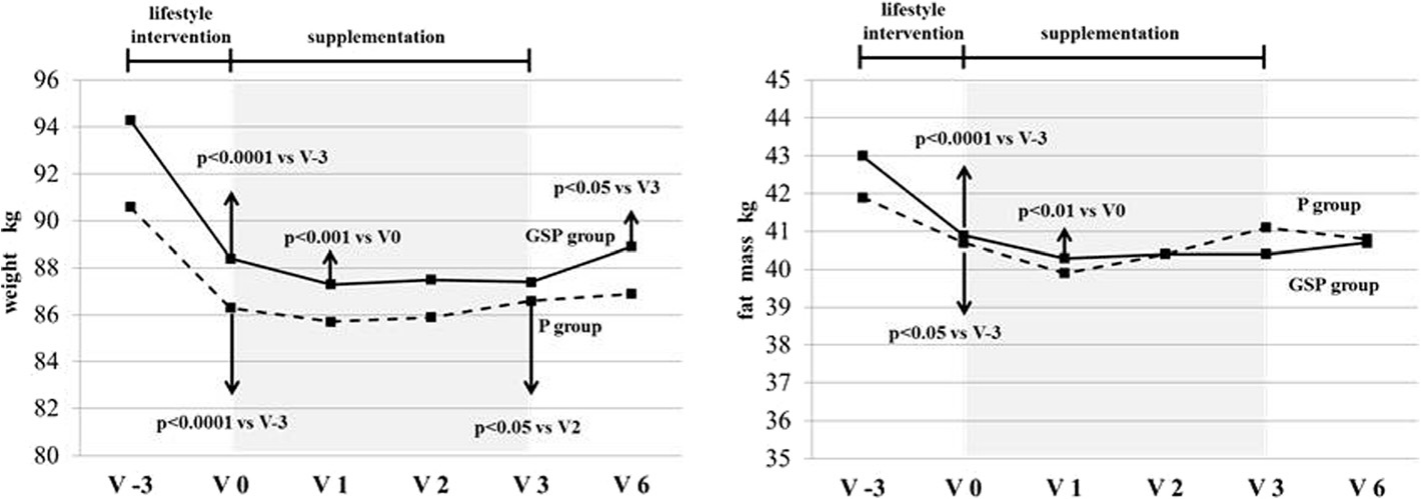
Weight (left panel) and fat mass (right panel) in GSP group (solid line) and P group (dashed line) at V-3, 0, 1, 2, 3 and 6; p values indicate differences between values at a defined visit and the preceding visit within each group. If the difference is not significant, the p value is not indicated

Changes in weight and fat mass during the study period are depicted in Fig. 3. At the first month of dietary supplements (V1), the GSP group showed a further reduction in weight and fat mass that remained stable at V2 and V3 and increased three months after discontinuation of the supplements (V6). In the P group, weight and fat mass started to increase at V2. Weight change from V0 toV3 was −1.0 kg (95 % CI −2.5 to +0.5) in the GSP group and + 0.3 kg (95 % CI −0.9 to +1.6) in the P group.

The adherence to the diet progressively decreased in both groups from V0 to V6. Scores in GSP group were 5.4 ± 3.9 at V3, 2.2 ± 1.3 at V0 (*p* < 0.05 for V3 vs. V0), and 6.4 ± 4.9 at V6 (*p* = NS for V3 vs. V6). The scores in P group were 5.2 ± 4.0 at V3, 2.8 ± 1.6 at V0 (*p* < 0.05 for V3 vs. V0), and 7.8 ± 3.3 at V6 (*p* < 0.05 for V3 vs. V6). Advices on physical activity were apparently followed in both groups because fat-free mass remained stable from V0 to V6: scores in GSP group were 54.1 ± 3.3 % at V3, 53.8 ± 2.7 % at V0 (*p* = NS for V3 vs. V0), and 54.6 ± 3.8 % at V6 (*p* = NS for V0 and V3). In the P group, the scores were 52.6 ± 2.9 at V3, 53.0 ± 2.1 at V0 (*p* < 0.05 for V3 vs. V0) and 53.2 ± 5.1 at V6 (*p* < 0.05 when compared to V0 and V3).

Blood pressure and heart rate remained stable from V0 to V6 in both groups (data not shown).

**Fig. 4:**
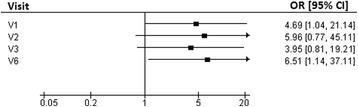
Effect of Greenselect Phytosome® compared to placebo on the probability to maintain a ≥5 % weight loss at V1, 2, 3 and 6

The proportion of obese women who maintained a weight loss ≥ 5 % was greater in the GSP than in the P group (75 % vs. 45 % at V1 and 60 % vs. 30 % at V6, *p* < 0.05 for both). The logistic regression analysis demonstrated a significantly higher probability to maintain a ≥5 % weight loss at V1 and V6 in the GSP group than in the P group (Fig. 4).

## Discussion

Results of this study indicate that after a weight loss intervention, obese women supplemented with GSP are more likely to maintain the reduction in body weight achieved with the diet than those receiving placebo. Indeed, we observed that weight and fat mass decreased during the first month of dietary supplements with GSP and then remained stable during the period of supplement intake despite the decrease in the adherence to the diet. Conversely, the weight and fat mass of women belonging to the P group started to rise one month after the end of the lifestyle intervention. The trend to return to the pre-body fatness is an expected event after successful weight loss because of the occurrence of coordinate actions of behavioral, metabolic, neuroendocrine, and autonomic responses that are designed to maintain body energy stores [2]. GT has been shown to stimulate daily energy expenditure by 4–4.8 % [10, 11], particularly under conditions of elevated sympathetic tone [12] such as obesity. GSP may be therefore more useful to offset the reduction in energy expenditure caused by the adaptive thermogenesis when given in association with a weight loss program. In fact, during a low calorie diet and adequate physical activity, the increase in the daily energy expenditure induced by GSP might not be effective enough due to the overriding effects of the initial changes in diet and physical activity. This finding is likely to explain the modest effects of GT on weight loss reported in the majority of randomized intervention studies [4, 13].

Few studies reported the effects of GT extracts on weight maintenance after a period of weight loss in overweight and moderately obese subjects. In a study by Kovasc et al., a 4-week weight loss period with a very low caloric diet was followed by a 13-week weight maintenance period in which, the subjects consumed their habitual diet and received GT-caffeine mixture or placebo. No significant differences in body weight regain were observed between the GT and placebo group however, a higher weight regain was observed in high caffeine consumers [14]. In another study, the same group confirmed that GT–caffeine mixtures may induce further weight reductions during the weight maintenance period only in low-level caffeine consumers [15]. These studies are not comparable to our study that utilized a GSP preparation devoid of caffeine, but with active polyphenols complexed with soy phospholipids and piperine to enhance bioavailability and stimulate thermogenesis.

We observed that the effect of Globes® on body fatness disappears after its discontinuation. This pattern does not seem be due to a lower adherence to lifestyle recommendations, because in GSP group the adherence to the diet and fat-free mass (proxy of physical activity) were similar between the end of the intake of dietary supplements and follow-up. If we consider that the decline of energy expenditure occurring after weight loss persists for at least one year [2], it can be assumed that GSP preparations should be taken for several months after the intensive phase of a diet to favor weight maintenance. This remains a hypothesis because energy expenditure was not assessed in this study. Future research having energy expenditure and respiratory quotient as primary endpoints would perhaps shed light on the mechanisms by which Globes® affect body fatness.

Finally, we emphasize that dietary supplements with Globes® did not affect blood pressure and heart rate levels, suggesting that the increase in sympathetic activity induced by GSP was not associated to significant cardiovascular changes.

## Conclusion

We believe that GSP extracts devoid of caffeine may have a clinical potential for the maintenance of weight after the intentional weight loss. More prolonged randomized studies are needed to confirm these effects and evaluate the dose of GSP required to achieve expected results.

## Abbreviations

BP, blood pressure; GSP, greenselect phytosome®; GT, green tea; MET, metabolic equivalent; P, placebo; V, visit

## Additional files

Additional file 1: Questionnaire for the evaluation of adherence to the diet. (DOCX 16 kb) Additional file 2: Table S1. Dietary composition and physical activity of obese women of GSP and P group before and after the 3-month lifestyle intervention. (DOCX 16 kb)
