# Supplementary material for: Effects of Greenselect Phytosome® on weight maintenance after weight loss in obese women: a randomized placebo-controlled study
Source: BMC Complement Altern Med. 2016 Jul 22;16:233. doi: 10.1186/s12906-016-1214-x (PMC4957378; doi:10.1186/s12906-016-1214-x)
Supplement: Additional file 1: — Questionnaire for the evaluation of adherence to the diet. (DOCX 16 kb) [file 12906_2016_1214_MOESM1_ESM.docx]

Additional file 1: Questionnaire for the evaluation of adherence to the diet

| 1. Cereals   (serving/day) |  0   1   2 | concordant to dietary prescription  slightly discordant from dietary prescription  discordant from dietary prescription |
| --- | --- | --- |
| 1. Fruits and vegetables   (serving/day) |  0   1   2 | concordant to dietary prescription  slightly discordant from dietary prescription  discordant from dietary prescription |
| 1. Meat / fish /eggs / legumes   (serving/week) |  0   1   2 | concordant to dietary prescription  slightly discordant from dietary prescription  discordant from dietary prescription |
| 1. Dairy products   (serving/day) |  0   1   2 | concordant to dietary prescription  slightly discordant from dietary prescription  discordant from dietary prescription |
| 1. Seasoning and high fat foods   (serving/day) |  0   1   2 | concordant to dietary prescription  slightly discordant from dietary prescription  discordant from dietary prescription |
| 1. Sweets and soft drinks   (serving/week) |  0   1   2 | concordant to dietary prescription  slightly discordant from dietary prescription  discordant from dietary prescription |
| 1. Alcoholic beverages   (serving/day) |  0   1   2 | never  1 glass of wine (125ml) or beer (330ml)  ≥2 glasses of wine or beer |
| 1. Snacking   (number/week) |  0   1   2 | ≤1  2-3  >3 |
| 1. Gluttony |  0   1   2 | 0/week  ≤4 times/week  >4 times/week |

Score: 0-4 = good adhesion to diet; 5-10 = fairly good adhesion to diet; 11-18 = poor adhesion to diet
