# Supplementary material for: Effects of Greenselect Phytosome® on weight maintenance after weight loss in obese women: a randomized placebo-controlled study
Source: BMC Complement Altern Med. 2016 Jul 22;16:233. doi: 10.1186/s12906-016-1214-x (PMC4957378; doi:10.1186/s12906-016-1214-x)
Supplement: Additional file 2: Table S1. — Dietary composition and physical activity of obese women of GSP and P group before and after the 3-month lifestyle intervention. (DOCX 16 kb) [file 12906_2016_1214_MOESM2_ESM.docx]

Additional file 2: Table S1 Dietary composition and physical activity of obese women of GSP and P group before and after the 3-month lifestyle intervention

|  | GSP group (n=20) | | P group (n=20) | |
| --- | --- | --- | --- | --- |
|  | V-3 | V0 | V-3 | V0 |
| Energy, kcal/day | 2039±504 | 1639±317** | 1715±342§ | 1463±291* |
| Saturated fat, % energy | 9.1±3.2 | 6.5±1.8* | 8.0±2.3 | 7.2±2.0 |
| Monounsaturated fat, % energy | 14.6±4.6 | 11.6±2.5** | 13.1±4.1 | 13.4±3.8 |
| Polyunsaturated fat, % energy | 3.2±0.7 | 2.7±0.5** | 3.0±0.7 | 3.3±0.8 |
| Animal protein, % energy | 11.3±4.2 | 10.2±4.3 | 10.1±3.5 | 9.9±4.7 |
| Vegetable protein, % energy | 6.7±1.6 | 7.6±1.9 | 7.2±1.6 | 8.1±1.3 |
| Soluble carbohydrates, % energy | 16.5±4.9 | 16.8±4.2 | 14.7±3.7 | 15.2±3.3 |
| Starch carbohydrates, % energy | 30.9±7.9 | 34.1±8.7 | 35.6±7.2 | 32.9±6.8 |
| Physical activity (MET, minutes/week)^a^ | 600(219-1188) | 1596(1331-2938)*** | 519(297-1557) | 1731(1240-2865)*** |

V-3: before lifestyle intervention. V0: after 3-month lifestyle intervention-start of supplementation.

^a^ Expressed as median (interquartile range)

§ p<0.05 vs GSP group; *p<0.05, **p<0.01, ***p<0.0001 vs V-3
